# First case report of long-term latent infection paracoccidioidomycosis in China

**DOI:** 10.1097/MD.0000000000041409

**Published:** 2025-02-14

**Authors:** Haiping Dong, Jingyuan Feng, Shaoling Wu, Feng Liang, Hua Li, Xiaocheng Liang, Weixiang Liao, Yanshan Pan, Guidan Tang, Donghai Li, Wei Zhou, Zhizhong Cao, Weiyong Wang, Jinxing Hu

**Affiliations:** a The Second Tuberculosis Department, Guangzhou Chest Hospital, Guangzhou, Guangdong, China.

**Keywords:** lymphadenectasis, next-generation sequencing, *Paracoccidioides*, paracoccidioidomycosis

## Abstract

**Rationale:**

Although paracoccidioidomycosis is one of the most prevalent endemic mycoses in Latin American countries, where at least 10 million people are infected, the prevalence of paracoccidioidomycosis in China remains unknown because no related case has been reported, and its diagnosis is extremely challenging for local clinicians because of the complexity of disease progression and lack of specific evidence.

**Patient concerns:**

Here, we report the first case of PCM in a male patient with a long-term latent infection in China.

**Diagnosis:**

The results of special staining, immunohistochemistry, lymph node biopsy pathology, and metagenomic second-generation sequencing indicated that the patient was infected with *Paracoccidioides brasiliensis*.

**Interventions:**

In this case, the patient was administered voriconazole 200 mg twice daily.

**Outcomes:**

After continuous treatment for 6 months, the patient’s symptoms improved significantly, and the medication was discontinued. The outpatient follow-up revealed no discomfort.

**Lessons:**

This case is of great value for the early diagnosis, treatment, and prevention of the spread of this emerging infectious disease in China.

## 1. Introduction

Paracoccidioidomycosis was initially reported in 1908 in São Paulo, Brazil by Adolpho Lutz, and is primarily a systemic and neglected tropical mycosis.^[1]^ In areas with high epidemic rates, such as Brazil, the annual incidence of paracoccidioidomycosis reaches 3 cases per 100,000 inhabitants, and the fatality rate is 2% to 23%.^[2]^ Systemic mycosis is the most common cause of mortality in Brazil (Table S1, Supplemental Digital Content, http://links.lww.com/MD/O334).^[3]^

Paracoccidioidomycosis is caused by the fungus *Paracoccidioides* spp., which is endemic in Latin America (Table S1, Supplemental Digital Content, http://links.lww.com/MD/O334).^[1,4]^ These fungi live in parts of Central and South America, and are thermally dimorphic fungi that switch from mycelial or conidia forms found in the environment at 26 °C to a yeast form during mammalian infection at 37 °C.^[5]^ Anyone who lives in or visits areas where *Paracoccidioides* lives can develop paracoccidioidomycosis, but it most often affects men who work outdoors in rural areas.^[6]^ The primary infection site is the lungs, as inhalation of conidia or mycelial propagules is the most common manner of transmission.

Although paracoccidioidomycosis is one of the most prevalent endemic mycoses in Latin American countries where at least 10 million people are infected, the prevalence of paracoccidioidomycosis in China remains unknown since no related case have been reported, and its diagnosis is extremely challenging for local clinicians because of the complexity of disease progression and lacking of specific evidence. Here, we report the first case of PCM in a male patient with long-term latent infection in China.

## 2. Materials and methods

### 2.1. Medical history and clinical data

**Figure 1.**
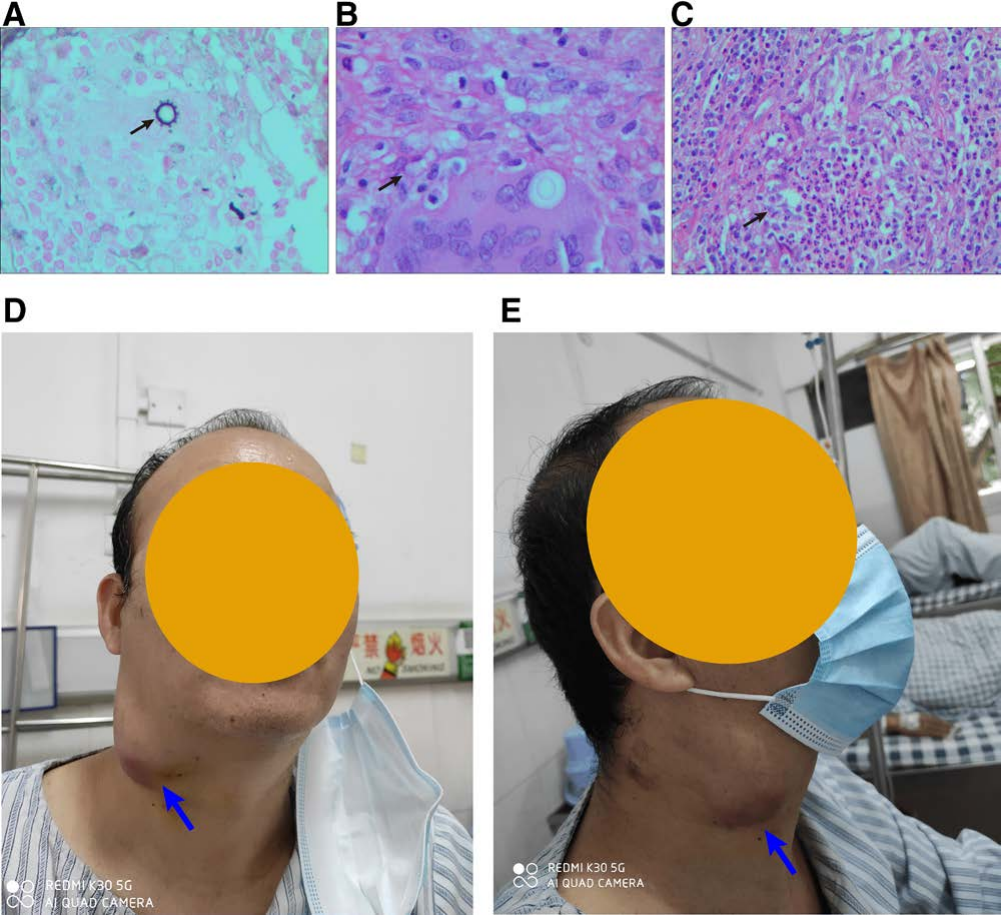
Pathogenic detection and clinical manifestations of the patient before hospitalization. Pathological microscopic images of lymph node biopsy (A–C) and photos of enlarged lymph nodes in the patient’s neck (D–E). The black arrows indicate chronic granulomatous inflammation with a small abscess formation. The blue arrows indicate the clinical manifestations.

The study was provided by the Medical Ethics Committee of Guangzhou Chest Hospital (approved number: Xiongyi Ethics [2022] No. 62). It was granted by ethics committee with the permission of retrospectively and anonymously assessing obtained surveillance data on our forces returning from deployment. The study has been conducted in accordance with the Declaration of Helsinki and all its amendments. Informed consent was obtained from the subject involved in the study. Written informed consent has been obtained from the patient to publish this paper. The patient, a 48-year-old male, was admitted to the hospital on February 24, 2021 due to the discovery of a neck tumor for >5 months. Five months before admission, the patient developed multiple bilateral neck masses without obvious causes, without pain, rash, or fever, and no further diagnosis or treatment was given. Three months prior to admission, the neck mass had increased in size. After admission to a hospital, the patient was performed a right neck mass resection surgery. Postoperative pathological tissue examination indicated chronic granulomatous inflammation with a small abscess formation (Fig. 1A–C), suggesting a high risk of tuberculosis. Therefore, the doctor performed anti-tuberculosis treatment with rifampicin, isoniazid, ethambutol, and moxifloxacin. One week later, drug-induced hepatitis occurred, and tuberculosis drugs were discontinued. Liver protection treatment was administered for 2 weeks, and the patient’s liver function improved. The patient’s right neck lymph nodes began to enlarge more than a month before admission. The patient was treated with isoniazid, ethambutol, and moxifloxacin again for tuberculosis until admission. Although the patient received regular antituberculosis treatment, the lymph nodes in the right neck still progressively enlarged. At admission, the largest lymph node was egg-sized (Fig. 1D and E), accompanied by tenderness, no rupture, no redness or swelling, and no fever.

The patient had a history of hepatitis B virus infection for more than 20 years and started antiviral treatment with entecavir 5 months prior to admission. The patient was born and lived in their home country. He had a history of traveling abroad and working as a restaurant chef in a Brazilian farm from 1993 to 2017. The patient had a smoking history for more than 20 years, had taken 20 cigarettes per day, and had quit smoking for 7 years upon admission. The patient had a history of alcohol consumption for >20 years, with a daily intake of 100 mL. At the time of admission, the patient had abstained from drinking for 1 year.

When the patient was admitted, his vital signs were stable, consciousness was clear, and multiple swollen lymph nodes could be palpated on both sides of the neck. The right side was obviously enlarged, with the maximum mass of about 4.7 × 3.7 cm with a hard texture, tenderness, no wave sensation, no redness or swelling, and moderate mobility (Fig. 1D). The patient’s armpit and groin did not show any enlarged lymph nodes.

### 2.2. Examination and treatment

After the patient was admitted, serum hepatitis B surface antigen, anti-HCV, HIV virus antibody and antigen, *Treponema pallidum* antibody, and rapid plasma regain were tested using chemiluminescence. Serum fungal levels of 1–3-β-D-glucan and G-lipopolysaccharide I were determined using the colorimetric method. Serum hypersensitive C-reactive protein was tested using immunofluorescence. Serum procalcitonin was measured as previously described.^[7]^

The specific cytokines of *Mycobacterium tuberculosis* were tested using the *M tuberculosis*-specific cytokine detection reagent (Deaou, Guangzhou, Guangdong, China). *M tuberculosis* was cultivated using a MGIT 960 fully automatic analyzer (BD, Franklin Lakes, NJ). The drug sensitivity of the pathogenic bacteria was determined using the VITEK compact microbial identification system (BioMérieux, Durham, NC) and a VITEK MS spectrometer (BioMérieux, Durham, NC). *Aspergillus* was tested using a commercial kit (Bio-Rad, Hercules, CA). CD4/CD8/CD3 lymphocyte subpopulations were tested using a BEION M4‐ BF biological microscope (Beionmed, Shanghai, China) as previously described.^[8]^ The interferon gamma release assay (IGRAS) was conducted using the *M tuberculosis*-specific cellular immune response assay kit (Signature Biotechnology, Foshan, Guangdong, China). Lymph node biopsy was performed for *M tuberculosis* nucleic acids using a Gene Xpert MTB/RIF kit (Cepheid, CA). *Aspergillus* antigen (GM test) in bronchoalveolar lavage fluid was tested using an ELISA kit. Acid-resistant bacteria were tested using smears. Nasopharyngeal laryngoscopy and bronchoscopy were performed using an electronic nasopharyngolaryngoscope (Olympus, Japan). Neck computed tomography (CT) was conducted using an Aquilion 16-row multislice CT (Canon, Japan) with 120 kV tube voltage, 100 to 400 mA tube current, 1 pitch, 3 mm layer thickness, 3 mm layer spacing, 512 to 512 of matrix, and 3 mm recombination interval. Enhanced scanning for 20 to 30 seconds (arterial phase) and 60 to 70 seconds (venous phase) and intravenous infusion of iodofoxol (Jiangsu Hengrui Pharmaceutical Co., Ltd., Lianyungang, Jiangsu, China) at a flow rate of 3.0 mL/s through a pump was performed to obtain axial, sagittal, and coronary MPR images, with a reconstituted thickness of 3 mm. The patient was on an empty stomach for >4 hours, with stable blood sugar, and then intravenously injected with 18F-FDG, and after resting for approximately 60 minutes. Subsequently, whole-body positron emission tomography-CT was performed at the Guangzhou Huyun Medical Imaging Diagnostic Center (Guangzhou, China). Metagenomic next-generation sequencing of lymph node biopsy was conducted on a HiSeq 3000 platform (Illumina Way, San Diego, CA) in GensKey (Beijing, China) as previously described.^[9,10]^

After admission, the patient continued to receive antituberculosis treatment. Two weeks later, he was diagnosed with paracoccidioidomycosis and began treatment with voriconazole on March 9, 2021; and isoniazid was retained for preventive treatment. The patient’s symptoms gradually improved and the medication was discontinued on August 31, 2021.

## 3. Results

The patient’s white blood cell, red blood cell, eosinophil, and platelet counts were 8.47 × 10^9^, 3.48 × 10^12^, 0.83 × 10^9^, and 210.0 × 10^9^ cells/L. The hemoglobin concentration was 107.0 g/L, and the percentage of eosinophils was 9.8% (Table 1). Pathogen detection results of the bronchial lavage were negative. Results of IGRAS, detections of specific cytokines, antibodies, C-reactive protein, and erythrocyte sedimentation rate, and skin test for purified protein derivative of tuberculin were negative. The result of sputum *M tuberculosis* tested by polymerase chain reaction was negative. Hepatitis B surface antigen result was positive. The patient’s CD4/CD8 lymphocyte subsets were normal, hypersensitive C-reactive protein was 26.74 mg/L, and procalcitonin was 0.07 mg/L (Table 1). Both bacterial and fungal cultures were negative.

**Table 1:** Patient clinical parameters and normal reference values.

Clinical parameter	Patient value	Normal reference value
WBC	8.47 × 10^9^ cells/L	4–10 × 10^9^ cells/L
RBC	3.48 × 10^12^ cells/L	3.5–5.5 × 10^12^ cells/L
EO	0.83 × 10^9^ cells/L	0.02–0.5 × 10^9^ cells/L
PC	210.0 × 10^9^ cells/L	100–300 × 10^9^ cells/L
HGB	107.0 g/L	120–160 g/L
EO%	9.8%	0.5–5%
hs-CRP	26.74 mg/L	0.06–10 mg/L
PCT	0.07 mg/L	≤ 0.05 mg/L

EO = eosinophils, EO% = percentage of eosinophils, HGB = hemoglobin concentration, hs-CRP = hypersensitive C-reactive protein, PC = platelet counts, PCT = procalcitonin, RBC = red blood cell, WBC = white blood cell.

**Figure 2.**
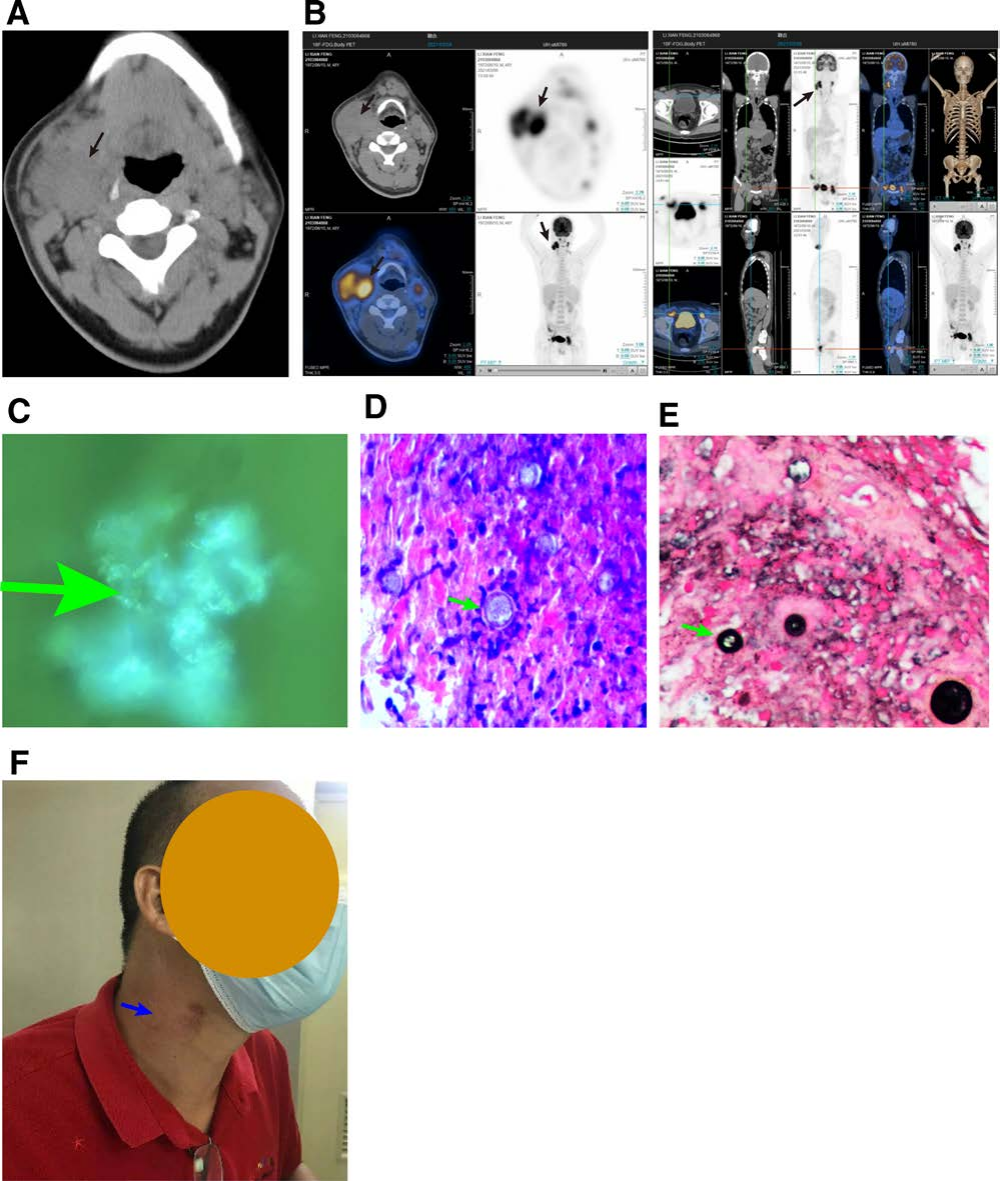
Imaging and pathogenic examination of the patient before treatment and the performance after treatment. (A) Neck CT; (B) PET-CT; (C) fungal fluorescence staining of lymph node biopsy tissue; (D) Schiff periodic acid Shiff staining; (E) periodic-acid silver methenamine staining; (F) Photo taken during follow-up after recovery. The black arrows indicate the imaging of the lesion. The green arrows indicate the results of pathogenic examination. The blue arrow indicates the lesion after treatment. CT = computed tomography, PET-CT = positron emission tomography-CT.

Nasopharyngoscopy and bronchoscopy revealed chronic pharyngitis without any other obvious abnormalities. Fungal culture and *Aspergillus* antigen detection in the infusion solution yielded negative results. The neck CT results showed multiple enlarged lymph nodes in the deep surface of the bilateral sternocleidomastoid muscle, bilateral carotid artery sheath area, neck root, and submandibular region, with uneven density and low-density areas inside. The edge of the lesion had a CT value of approximately 43 HU, with the largest one located in the shallow surface of the right sternocleidomastoid muscle, with a size of approximately 4.7 × 3.7 cm. The enhanced scan showed circular enhancement with a CT value of approximately 76 HU. The low-density areas inside were not enhanced and were partially fused, and the subcutaneous soft tissue was slightly swollen. Adjacent to the right side, the sternocleidomastoid muscle and carotid sheath were compressed and displaced inwards (Fig. 2A). Positron emission tomography-CT results showed that the patient had multiple enlarged lymph nodes, partially fused into clusters, and had active metabolism. The patient’s tonsils were enlarged and metabolically active bilaterally, with the right side being the most prominent. The patient had a nodular metabolic activity in the right lower abdominal ileocecal region. These lesions suggested multiple infiltrations inside and outside the lymphoma lymph nodes. There were slightly enlarged lymph modes in the hepatic hilum, abdominal aorta, and abdominal mesentery, with no metabolic abnormalities. Inflammatory hyperplasia was the first consideration. There were slightly enlarged lymph nodes in the hepatic hilum, abdominal aorta, and abdominal mesentery, with no metabolic abnormalities. Inflammatory hyperplasia was the first consideration (Fig. 2B). Semar acid-fast staining of lymph node biopsy tissue was negative, whereas fungal fluorescence staining was positive (Fig. 2C). Both periodic acid silver methenamine and Schiff periodic acid Shiff staining showed large and round cell walls with a double-layer structure and a strong alkaline spore structure (Fig. 2D and E). Transparent spore structure with empty cell wall within spore body. Immunohistochemical analysis showed positive CD68 epithelial cells and negative BCG. Based on the HE morphology and special staining results, the lesion was consistent with coccidiosis. A total of 49,795,725 reads were obtained from mNGS of lymph mode biopsy tissue, of which 423,020 reads were from nonhuman sources, with a sequencing accuracy of 99.70%. A total of 1130 reads were matched to *Paracoccidioides*, of which 803 reads were matched to *Paracoccidioides brasiliensis*. Since the patient worked as a restaurant chef in a Brazilian farm from 1993 to 2017, had a history of travel to Brazil, had enlarged lymph nodes throughout the body, and pathological support for the disease was consistent with paracoccidioidomycosis. Therefore, the final diagnosis was disseminated paracoccidioidomycosis.

Voriconazole is used to treat mild-to-moderate paracoccidioidomycosis.^[2]^ The patient in this case was treated with voriconazole at a dose of 200 mg twice daily when diagnosed with disseminated paracoccidioidomycosis. After continuous treatment for 6 months, the patient’s symptoms improved significantly, and the medication was discontinued. There was no discomfort during the outpatient follow-up (Fig. 2F).

## 4. Discussion

Paracoccidioidomycosis can cause a chronic suppurative granulomatous disease in humans with a chronic progressive course that preferentially affects the lungs, followed by the skin, mucous membranes, adrenals, and reticuloendothelial organs.^[5]^ One of the main risk factors for paracoccidioidomycosis is exposure to soil in rural areas, which is an occupational disease among farmers in endemic areas.^[11,12]^ Affected areas tend to shift from rural to urban areas. Paracoccidioidomycosis has been reported in various countries in South America, except for Chile and Guyana. Although pathogenic fungi are geographically limited to various Latin American countries,^[13]^ paracoccidioidomycosis cases have been reported in Japan^[14]^ and the USA.^[15]^ However, paracoccidioidomycosis has not been reported in China to date. Here, we reported a case of paracoccidioidomycosis in China that was misdiagnosed as tuberculosis.

Although the source of infection for paracoccidioidomycosis remains unclear, it is widely believed that the invasion of pathogenic fungi into the human body through oral, skin, and mucosal damage is a triggering factor for this disease. Paracoccidioidomycosis is predominant in adult males. However, this sex difference has not been observed in children or adolescents.^[5]^

Paracoccidioidomycosis is classified into mucocutaneous, pulmonary, and disseminated types according to their clinical manifestations. For the mucocutaneous type, pathogenic fungi often enter the human body through the oral mucosa or skin, and lesions initially occur in the gums, oral cavity, upper jaw, throat, nasal cavity, lips, and tongue. In the early stages of the lesion, nearby lymph nodes become enlarged, especially in the neck, which can become hard but not painful. Subsequently, local necrosis, suppuration, and perforation of the skin can occur to form fistulas. In the pulmonary type, *P brasiliensis* can reach many tissues, whereas it mainly attacks the lungs.^[16]^ The lungs are often involved, and their clinical manifestations must be differentiated from those of many other infectious and noninfectious diseases.^[17]^ Pulmonary infections are mostly primary, and primary pulmonary infections are often caused by the inhalation of spores. For the disseminated type, the primary infection spreads through the blood to the liver, spleen, small intestine, urinary and reproductive systems, central nervous system, muscles, bones, and organs, causing granulomas and purulent modules. The lymph modes throughout the body can also be affected and enlarged. Lymph nodes in the abdominal cavity can become enlarged, which easily are mistaken for tuberculosis or tumors. If lymph nodes are enlarged throughout the body, the disseminated paracoccidioidomycosis is often misdiagnosed as Hodgkin lymphoma.^[15]^ Severe lesions can spread to the central nervous system, urogenital system, muscles, cartilage, and other organs. Mucocutaneous paracoccidioidomycosis should be distinguished from cutaneous tuberculosis and tumors. Disseminated paracoccidioidomycosis should be distinguished from kala-azar, tuberculosis, lymphoma, and tumors.

The pathogenic fungus of paracoccidioidomycosis (*Paracoccidiodes* spp.) is a biphasic fungus that can form a hyphal phase when grown at room temperature.^[18]^ Its growth rate is slow and it requires a long cultivation period of up to 30 days.^[19]^ In the laboratory, sputum, secretions, mucosal scrapes, and lymph mode extracts can be directly examined under a microscope, and single- or multi-bud thick-walled spores can be observed under a microscope. In clinical practice, fungal cultures in paracoccidioidomycosis are usually positive. Under a microscope, atypical small conidia are observed on both sides of the hyphae.^[1]^ Serum tests can be used to detect antibodies and antigens. Pathologically, it is mainly characterized by purulent granulomas that often present as single-bud spores. Paracoccidioidomycosis is usually diagnosed based on clinical manifestations, fungal examination, and discovery of characteristic pathogens in combination with tissue pathology.^[1]^

Azoles have good therapeutic efficacy for the treatment of paracoccidioidomycosis, and oral itraconazole is generally the first choice. Intravenous amphotericin B can also cure infection and is often used in patients with more severe paracoccidioidomycosis. As antifungal drugs, sulfonamides can improve the disease, but cannot cure it. Early discovery, diagnosis, and intervention are essential for patients with paracoccidioidomycosis. Otherwise, patients would have a poor prognosis, with a recurrence rate of approximately 15%. Delayed diagnosis may lead to severe complications such as pulmonary changes and even death.^[20]^ Death due to paracoccidioidomycosis is caused by several complications such as extremely disseminated lesions, respiratory dysfunction, and adrenal cortex dysfunction, which may occur for a long time after antifungal treatment.^[11]^

This case was initially misdiagnosed as lymph node tuberculosis and anti-tuberculosis treatment was ineffective. It is difficult to distinguish paracoccidioidomycosis from tuberculosis in several aspects. Most published cases of paracoccidioidomycosis were initially misdiagnosed as tuberculosis, sarcoidosis, or squamous cell carcinoma.^[21,22]^ Therefore, clinical management guidelines specifically focus on comparative analysis of paracoccidioidomycosis and tuberculosis.^[12]^ This case indicated that for patients with normal immune cell function, IGRAS negative, double negative in the *M tuberculosis*-specific cytokine test, and sputum bacteria without evidence of tuberculosis, the diagnostic conclusion needs to be reconsidered.

Because it was difficult to distinguish it from tuberculosis in many aspects, this case was first misdiagnosed as lymphatic tuberculosis with no response to anti-tuberculosis therapy. Most of the reported PCM cases were in male patients initially misdiagnosed as tuberculosis, sarcoidosis, or squamous cell carcinoma.^[21,22]^ The clinical management guidelines specifically propose a comparative analysis of paracoccidioidomycosis and tuberculosis.^[12]^ Therefore, the diagnosis should be reconsidered in patients with normal immune function who have no evidence of tuberculosis, such as IGRAS-negative, double-negative *M tuberculosis*-specific cytokine test, purified protein derivative negative, and no evidence of tuberculosis in sputum cultures.

Serological tests are widely used in the presumptive diagnosis and follow-up of cases of paracoccidioidomycosis.^[12,23]^ This case indicated that conducting serological tests based on foreign travel history and the pathogens of infectious diseases experienced in the region was crucial in clinical diagnosis.

## 5. Conclusions

This case indicated that (1) if there is no clear etiological basis for tuberculosis, and if the effect of anti-tuberculosis treatment is not good, it is necessary to consider the possibility of misdiagnosis and make further diagnosis in time. Therefore, attention should be paid to NTM and fungal infections. (2) Compared to traditional detection methods, NGS has the advantages of comprehensiveness, sensitivity, and speed, making it of great significance for the diagnosis and treatment of rare pathogen infections. (3) New pathogen detection technologies should be properly utilized to achieve precise diagnosis and treatment.

## Acknowledgments

Jiajia Ni from Guangdong Meilikang Bio-Science Ltd. (China) is gratefully acknowledged for his support with data management.

## Author contributions

**Conceptualization:** Haiping Dong, Weiyong Wang, Jinxing Hu.

**Data curation:** Donghai Li, Wei Zhou, Zhizhong Cao.

**Formal analysis:** Haiping Dong, Jingyuan Feng, Shaoling Wu, Feng Liang.

**Funding acquisition:** Haiping Dong, Jinxing Hu.

**Investigation:** Haiping Dong, Feng Liang, Hua Li, Xiaocheng Liang, Weixiang Liao, Yanshan Pan, Guidan Tang, Donghai Li.

**Methodology:** Haiping Dong, Jingyuan Feng, Jinxing Hu.

**Project administration:** Jinxing Hu.

**Resources:** Haiping Dong, Weiyong Wang, Jinxing Hu.

**Software:** Haiping Dong, Shaoling Wu.

**Supervision:** Wei Zhou, Zhizhong Cao.

**Validation:** Donghai Li, Wei Zhou, Zhizhong Cao.

**Visualization:** Haiping Dong, Jingyuan Feng.

**Writing – original draft:** Haiping Dong.

**Writing – review & editing:** Jinxing Hu.

## Supplementary Material


