# Supplementary material for: First case report of long-term latent infection paracoccidioidomycosis in China
Source: Medicine (Baltimore). 2025 Feb 14;104(7):e41409. doi: 10.1097/MD.0000000000041409 (PMC11835094; doi:10.1097/MD.0000000000041409)
Supplement: Supplementary file 1 [file medi-104-e41409-s001.pdf]

Supplementary materials

Table S1. Epidemic situation of paracoccidioidomycosis. There are too many cases in South America to list them all.

Pathogen	Country	Year of identification	Year of travel of the endemic country	Therapeutic strategy	Reason for the trip	Length of stay in the endemic country	Reference
Paracoccidioides brasiliensis S1	Brazil			Support treatment			[1]
Paracoccidioides brasiliensis PS2	Brazil			Sulfamethoxazole/trimethoprim (SMZ/TMP 800/160 mg b.i.d)			[2]
Paracoccidioides brasiliensis	USA	2017	2008	Oral itraconazole 200 mg twice a day	The patient is originally from Ecuador	The patient is originally from Ecuador	[3]
Paracoccidioides brasiliensis	Japan	2006	The patient had reportedly last returned to South America in 1994.	Initially administered amphotericin B lipid complex (200 mg/day) for 18 days. The patient then received itraconazole (300 mg/day) for 1 month. The patient continued treatment with itraconazole (200 mg/day) for about 10 months.	The patient is Argentinean.	unknown	[4]
Paracoccidioides spp.	Brazil			Surgery and itraconazole			[5]
Paracoccidioides lutzii	Brazil	2016		Sulfamethoxazole/trimethoprim 800/160 mg three times a day			[6]
Paracoccidioides	Brazil	1958-2021					[7]

spp.					
Paracoccidioides brasiliensis	Australian	unknown	Leave Brazil for three years	Oral itraconazole at 150 mg twice daily (suprabioavailable) and completed a total treatment duration of 12 months with therapeutic levels maintained throughout.	[8]
Paracoccidioides spp.	Colombia			Amphotericin B for 7 days and then Itraconazole 200 mg QD for 12 months.	[9]
Paracoccidioides brasiliensis	Brazil			Intravenous amphotericin B	[10]
Paracoccidioides brasiliensis	Brazil				[11]

References

1. de Macedo PM, Almeida-Paes R, de Abreu Almeida M, Coelho RA, de Oliveira Filho MA, Medeiros DM, Gomes-Silva A, de Lima JR, Da-Cruz AM, Zancopé-Oliveira RM, do Valle ACF. 2018. Fatal septic shock caused by *Paracoccidioides brasiliensis* phylogenetic species S1 in a young immunocompetent patient: a case report. Rev Soc Bras Med Trop 51, 111-114.
2. de Macedo PM, Almeida-Paes R, de Medeiros Muniz M, Oliveira MME, Zancopé-Oliveira RM, Costa RLB, do Valle ACF. 2016. Paracoccidioides brasiliensis PS2: First autochthonous paracoccidioidomycosis case report in Rio de Janeiro, Brazil, and literature review. Mycopathologia 181, 701-708.
3. Ghani A, Weinberg M, Pathan N, Vidhum R, Sieber S. 2018. Paracoccidioides brasiliensis infection mimicking recurrent Hodgkin Lymphoma: a case report and review of the literature. Mycopathologia 183: 973-977.
4. Sunada LT, Jinbu Y, Terauchi Y, Hayasaka J, Itoh H, Kusama M. 2008. Chronic paracoccidioidomycosis in Japan. Asian J Oral Maxillofac Surg 20, 89-93.
5. da Cruz ER, Forno AD, Pacheco SA, Bigarella LG, Ballotin VR, Salgado K, Freisbelen D, Michelin L, Soldera J. 2021. Intestinal paracoccidioidomycosis: Case report and systematic review. Braz J Infect Dis 25, 101605.

6. Tatagiba LS, Pivatto LB, Faccini-Martínez ÁA, Peçanha PM, Velloso TRG, Gonçalves SS, Rodrigues AM, Gamargo ZP, Falqueto A. 2018. A case of paracoccidioidomycosis due to *Paracoccidioides lutzii* presenting sarcoid-like form. Medical Mycology Case Reports 19, 6-8.
7. de Oliveira LLC, de Arruda JAA, Marinho MFP, Cavalcante IL, Abreu LG, Abrahão AC, Romañach MJ, de Andrade BAB, Agostini M. 2023. Oral paracoccidioidomycosis: a retrospective study of 95 cases from a single center and literature review. Med Oral Patol Oral Cir Bucal 28, e131-9.
8. Badrick TC, Meumann EM, Shirley K, Simos P, May ML, Quagliotto G, Bursle EC, Leonard N, McDougall RJ, Robson JM. 2024. Paracoccidioidomycosis: an Australian case. Med J Aust 220, 505-506.
9. Ramos DA, Alzate JA, Montoya ÁMG, Trujillo YA, Ramos LYA. 2020. Thinking in paracoccidioidomycosis: a delayed diagnosis of a neglected tropical disease, case report and review of clinical reports and eco-epidemiologic data from Colombia since the 2000. BMC Infectious Diseases 20, 119.
10. Sousa JAB, Sá RS, Pereira EM. 2021. Consequences of late diagnosis paracoccidioidomycosis: case report. J Bras Patol Med Lab 57, 1-3.
11. de Albuquerque Neto AD, Araújo AVA, Cerqueira DA, De Angeli Cesconetto L, Provenzano N, de Oliveira EMF. 2018. Diagnosis and treatment of paracoccidioidomycosis in the maxillofacial region: A report of 5 cases. Case Reports in Otolaryngology 2018, 1524150.
